# The impact of physiological noise correction on fMRI at 7 T

**DOI:** 10.1016/j.neuroimage.2011.04.018

**Published:** 2011-07-01

**Authors:** C. Hutton, O. Josephs, J. Stadler, E. Featherstone, A. Reid, O. Speck, J. Bernarding, N. Weiskopf

**Affiliations:** a Wellcome Trust Centre for Neuroimaging, UCL Institute of Neurology, University College London, London, UK; b Special Lab Non-Invasive Brain Imaging, Leibniz Institute for Neurobiology, Magdeburg, Germany; c Department of Biomedical Magnetic Resonance, Institute for Experimental Physics, Otto-von-Guericke University, Magdeburg, Germany; d Institute for Biometry and Medical Informatics, Faculty of Medicine, Otto-von-Guericke University, Magdeburg, Germany

**Keywords:** Physiological noise, SNR, Temporal SNR, tSNR, fMRI, 7 T

## Abstract

Cognitive neuroimaging studies typically require fast whole brain image acquisition with maximal sensitivity to small BOLD signal changes. To increase the sensitivity, higher field strengths are often employed, since they provide an increased image signal-to-noise ratio (SNR). However, as image SNR increases, the relative contribution of physiological noise to the total time series noise will be greater compared to that from thermal noise. At 7 T, we studied how the physiological noise contribution can be best reduced for EPI time series acquired at three different spatial resolutions (1.1 mm × 1.1 mm × 1.8 mm, 2 mm × 2 mm × 2 mm and 3 mm × 3 mm × 3 mm). Applying optimal physiological noise correction methods improved temporal SNR (tSNR) and increased the numbers of significantly activated voxels in fMRI visual activation studies for all sets of acquisition parameters. The most dramatic results were achieved for the lowest spatial resolution, an acquisition parameter combination commonly used in cognitive neuroimaging which requires high functional sensitivity and temporal resolution (i.e. 3 mm isotropic resolution and whole brain image repetition time of 2 s). For this data, physiological noise models based on cardio-respiratory information improved tSNR by approximately 25% in the visual cortex and 35% sub-cortically. When the time series were additionally corrected for the residual effects of head motion after retrospective realignment, the tSNR was increased by around 58% in the visual cortex and 71% sub-cortically, exceeding tSNR ~ 140. In conclusion, optimal physiological noise correction at 7 T increases tSNR significantly, resulting in the highest tSNR per unit time published so far. This tSNR improvement translates into a significant increase in BOLD sensitivity, facilitating the study of even subtle BOLD responses.

## Highlights

► Impact of physiological noise correction on tSNR versus SNR was characterized at 7 T. ► tSNR was improved by 50 to 70% using physiological noise correction in task-free EPI. ► The reported results exceed values for tSNR per unit time published so far. ► tSNR improvements translated into more than 10% increase in BOLD activity in fMRI.

## Introduction

In cognitive fMRI studies experimentally induced BOLD signal changes are often just a few tenths of a percent and therefore require maximal sensitivity and high temporal signal-to-noise ratio (tSNR) for reliable detection. The SNR and hence the tSNR can be increased by using higher static magnetic fields (B_0_). However, the relative contribution of physiological noise, including cardio-respiratory effects and head movement also increases with increased SNR (Kruger and Glover, 2001; Triantafyllou et al., 2005). Consequently strategies for reducing the effects of physiological fluctuations are particularly important in fMRI studies at higher field strengths such as 7 T.

If the subject moves during a scan this leads to an increase in signal variance which can also give rise to false positive activations if the motion is correlated with the task (Friston et al., 1996; Hajnal et al., 1994). The thoracic and abdominal movements involved in respiration result in head motion and modulation of the magnetic field leading to reproducible signal fluctuations as the subject breathes (Glover et al., 2000). Cardiovascular processes leading to pulsation in blood and cerebrospinal fluid give rise to periodic signal fluctuations linked to the cardiac cycle (Dagli et al., 1999; Glover et al., 2000). Since the cardiac and respiratory cycles (~ 1 s and ~ 3 s respectively) are often under-sampled relative to the EPI acquisition (repetition times are usually greater than 2 s), aliased signal fluctuations also occur in the fMRI time series with lower frequencies than the original physiological effects. Furthermore, lower frequency fluctuations in BOLD contrast have been reported to arise from changes in blood flow and CO_2_ levels associated with changes in breathing depth and rate (Birn et al., 2006; Wise et al., 2004). Additionally, a relation has been proposed between low frequency signal fluctuations and changes in heart rate which give rise to changes in blood oxygenation (Shmueli et al., 2007). These low frequency noise components are especially problematic since they often overlap with the frequencies of the experimental effects of interest.

The most widely accepted approach for reducing the effect of head motion is the retrospective realignment of image time series using image co-registration (e.g. Friston et al., 1995). Signal changes associated with motion that remain after the realignment procedure can be modeled as functions of the estimated realignment parameters (Friston et al., 1996). Signal fluctuations caused by the cardiac and respiratory cycles can be removed from the images using RETROICOR (Glover et al., 2000) or accounted for in the model used for statistical analysis (Josephs et al., 1997). This approach models cardiac and respiratory phases using peripheral measures of the subject's pulse and breathing. More recently studies have focused on the correction of lower frequency fluctuations related to changes in respiration and heart rate (Birn et al., 2006; Shmueli et al., 2007) and the development of impulse response functions to model these effects (Birn et al., 2008; Chang et al., 2009). Using these correction methods for fMRI data acquired at 3 T, improvement in the delineation of activity in the presence of task correlated physiological noise has been demonstrated (Birn et al., 2009). Furthermore, the corrections lead to a reduction in apparent false positive resting-state activations attributed to the default-mode network (Chang and Glover, 2009).

Given the SNR dependence of the physiological noise (Kruger and Glover, 2001; Triantafyllou et al., 2005), it is important to establish how well physiological noise correction methods work for fMRI at higher field strengths. It has been shown that by increasing the image resolution at 7 T, the corresponding reduction in image SNR reduces the relative contribution of physiological noise to the time series noise (Triantafyllou et al., 2005). Furthermore, tSNR was increased by acquiring high resolution fMRI data and spatially smoothing the images to lower resolutions (Triantafyllou et al., 2006). Many cognitive neuroimaging studies require fast whole brain image acquisition as well as maximal sensitivity to small BOLD signal changes. An important example is studies of the connectivity and causal interactions between different brain regions (e.g. den Ouden et al., 2009; Stephan et al., 2007) based on methods such as dynamic causal modeling (Friston et al., 2003), since they often assess interactions between remote brain areas using temporal signatures. At 7 T, we can achieve 3 mm isotropic resolution whole brain acquisitions in approximately 2–3 s even without speeding up image acquisition by using parallel imaging. In this study we therefore investigate the impact of physiological noise correction methods on this form of fMRI acquisition in addition to fMRI time series acquired at higher spatial resolutions.

One of our goals was to characterize the effect of physiological noise correction on tSNR as a function of image SNR. To do this we acquired task-free EPI time series and manipulated the image SNR by varying the radio-frequency (RF) excitation flip angles. We then applied physiological noise correction methods to the different time series and used an extension of the model proposed by Kruger and Glover(2001) to estimate a measure of the resulting tSNR degradation. Our second goal was to investigate the impact of the corrections on BOLD sensitivity for typical fMRI scenarios using standard analysis methods. For this we performed visual activation fMRI studies and compared the tSNR and the numbers of significantly activated voxels after applying different noise correction methods.

## Methods

### Data acquisition

We scanned a total of eleven healthy volunteers on a Siemens 7 T whole body MR scanner (Siemens Healthcare, Erlangen, Germany) using a 24-channel RF receive head coil with integrated CP volume RF transmit coil (Nova Medical, Inc., Wilmington, MA). Written informed consent was obtained from each participant for the study approved by the local Ethics committee. For six of the subjects, seven runs of EPI data were acquired. In five runs, we manipulated the image SNR by varying the RF excitation flip angle (see below under Temporal SNR study). During the other two runs we performed fMRI visual activation studies (see below under FMRI study). In one of the two fMRI runs, the subjects were instructed to produce task-correlated motion by moving their head synchronously with the stimulus. We do not present the data affected by motion here. Also, one of the first six subjects was excluded because a technical fault with the scanner shimming system made their data unusable. Finally, we scanned an additional five subjects performing the fMRI visual activation study as before but with two different sets of EPI acquisition parameters (see below under FMRI study).

The manufacturer's automatic adjustment procedure optimized for 7 T was performed at the beginning of each experiment to correct for first and second order distortions in the static magnetic field and to set the RF transmitter voltage. The EPI data were collected with three different sets of acquisition parameters which are given in Table 1. For all acquisitions the slice block was axial-to-coronal single oblique and aligned and centered manually with the calcarine fissure, and TE = 25 ms, volume TR = 2. For each subject, we also acquired an axial, dual echo, gradient echo field map and a T1-weighted anatomical image (MPRAGE, 1 mm isotropic resolution, TE = 3.72 ms, slice TR = 2000 ms, flip angle = 5°, inversion time TI = 1050 ms).

Throughout the EPI experiments we recorded the subjects' cardiac and respiratory signals and the scanner slice synchronization pulses using the Matlab Data Acquisition Toolbox (2009a, The MathWorks, Natick, MA) and a data acquisition device (NI USB-6009, National Instruments, Austin, Texas). The sampling rate was 100 Hz. The cardiac pulse signal was recorded from an MRI compatible pulse oximeter (Model 8600 F0, Nonin Medical, Inc. Plymouth, MN) attached to the subject's finger. The respiratory signal, thoracic movement, was monitored using a custom-made pneumatic belt positioned around the abdomen close to the diaphragm. The pneumatic pressure changes were converted into an analog voltage using a pressure transducer (Honeywell International Inc. Morristown, NJ) before digitization. The scanner slice synchronization pulses were recorded on one analog input after pulse shaping and lengthening for reliable detection.

### Temporal SNR study

For five of the EPI runs (acquired using Acq3 in Table 1), subjects were presented with a blank screen and instructed to rest with their eyes open to reduce the chance of falling asleep and so that their physiological state would be similar to that during the fMRI activation runs. Each run comprised 150 volumes and was acquired with one of the following flip angles: 8°, 16°, 26°, 38° and 70°, selected in a randomized order. We additionally acquired a thermal noise measurement of 20 EPI volumes with no RF excitation (i.e. 0° flip angle) (Triantafyllou et al., 2005). These flip angles produced images with an equally spaced range of SNR levels from 0° up to a maximum at 70°, the Ernst angle for gray matter at 7 T (assuming T_1_ = 1.9 s (Wright et al., 2008)).

### fMRI study

The fMRI stimulus was designed to alternately stimulate annular sectors of the left and right visual hemifields with contrast reversing black and white checkerboards. The contrast reversal rate was 8 Hz and each side was stimulated for 20 s (i.e. 10 EPI volume acquisitions) interspersed by presentation of the fixation screen for 20 s. This 80 second cycle (left; fixation; right; fixation) was repeated five times. Subjects were instructed to focus on a small, central fixation point which was present throughout the whole experiment.

Each experiment consisted of 205 volumes: five volumes acquired at the beginning of each run before starting stimulus presentation to allow for T_1_-related equilibration and for the subject to become accustomed to the scanning noise, followed by 200 volumes during stimulation. The EPI data were acquired using Acq3 with a flip angle of 70° in the first five subjects and using Acq1 and Acq2 with a flip angle of 80° in an additional five subjects.

### Data processing

All data were reconstructed using the scanner vendor's root sum-of-squares (rSoS) image reconstruction routines and then processed using SPM8 (http://www.fil.ion.ucl.ac.uk/spm/, Friston, 2007) with additional routines implemented in Matlab (2009a, The MathWorks, Natick, MA). After discarding the first five volumes of each run, the EPI data were spatially co-registered to the first volume of the first run. The gradient echo field map was processed to create a voxel displacement map and used to correct the realigned images for geometric distortion (Hutton et al., 2002). The following sections will describe the subsequent processing steps: definition of regions of interest (ROIs), physiological noise models, analysis of tSNR, comparison of physiological models at different spatial resolutions and analysis of fMRI studies.

### Definition of regions of interest

A visual cortex (VC) ROI covering a total volume of ~ 172 cm^3^ in MNI space (Evans et al., 1993), was defined anatomically according to the brain atlas provided with the AAL toolbox (Tzourio-Mazoyer et al., 2002). A lateral geniculate nucleus (LGN) ROI was defined in MNI space on the basis of a fixed effects group analysis of the spatially normalized fMRI data (with no physiological noise correction) acquired using Acq3 (i.e. with the largest voxel sizes). The following steps were performed to identify consistent LGN activity across the five subjects studied using Acq3. Each subject's anatomical image was registered to their corresponding undistorted, realigned EPI data and segmented into gray and white matter tissue probability maps using the unified segmentation procedure in SPM8 (Ashburner and Friston, 2005). The spatial normalization parameters resulting from this step were applied to the realigned and distortion corrected fMRI time series from each subject to transform the images into MNI space. The spatially normalized images were then smoothed using an isotropic Gaussian kernel with FWHM = 8 mm. A fixed effects general linear model (GLM) was constructed in SPM8 to perform a voxel-wise F-test for the effects of right and left visual stimulation over all of the subjects, together. The resulting map of F-statistics was thresholded at p < 0.05 (corrected for family-wise error using random field theory, Worsley et al., 1996) to yield a mask containing the significantly activated regions over the 5 subjects. As well as visual cortex this mask included clearly delineated regions in left and right LGN which were manually selected, smoothed and thresholded to create the LGN ROI (total volume of ~ 2.8 cm^3^ in the MNI space). Subject specific ROIs were next created by resampling the VC and LGN ROIs into the space of each subject using the inverse spatial normalization parameters resulting from the initial segmentation step. Finally the ROIs were restricted to gray matter by masking with the gray matter tissue probability map (from the initial segmentation step), thresholded at a value of p(GM) > 0.01. Note that the group fMRI analysis was performed only to identify the LGN ROI and not for the assessment of physiological noise correction which is described later. Furthermore an LGN ROI was constructed only for the fMRI data acquired using Acq3. The reduced brain coverage for the data acquired at higher spatial resolutions (i.e. Acq 1 and Acq2) meant that the LGN was not imaged in all subjects for these data sets.

### Physiological noise models

Four physiological noise models were constructed to account for artifacts related to 1) cardiac and respiratory phases (*CRP*), 2) respiratory volume (*RV*), 3) heart rate (*HR*), and 4) estimated head motion parameters (*MP*). Models for cardiac and respiratory phases and their aliased harmonics (*CRP*) were based on RETROICOR (Glover et al., 2000) and a similar, earlier method (Josephs et al., 1997). Rising edges were extracted from the measured cardiac peripheral pulse waveform to identify the time of each heart beat and the cardiac phase was estimated for each image slice (Glover et al., 2000). The respiratory waveform was sampled at the frequency of the scanner slice acquisition (i.e. at 20 Hz) and the respiratory phase at each time point was estimated as described in Glover et al.(2000). A basis set of sine and cosine Fourier series components extending to the 3rd harmonic (i.e. 6 terms) was used to model the fluctuations arising from the cardiac phase and was sampled at a reference slice in each image volume. The slice containing the calcarine fissure was selected as the reference slice so that the modeling was optimal in that part of the brain. Fluctuations arising from the respiratory phase were modeled in the same way. This resulted in a total of 12 model regressors containing a value for each time point. This component of the noise model is referred to as *CRP* (*for cardiac and respiratory phases*).

The lower frequency changes in respiration and heart rate (*RV* and *HR*) were modeled using an approach based on a combination of previously published methods (Birn et al., 2006; Birn et al., 2008; Chang et al., 2009; Shmueli et al., 2007). The implementation closely followed the method described in Chang and Glover(2009). A time series representing the change in respiration was calculated from the respiratory waveform by calculating the standard deviation at each time point over a 6 second sliding window. This was then convolved with the impulse response function for respiration (i.e. the ‘respiratory response function’) proposed in Birn et al.(2008) and sampled to the selected reference slice. The resulting regressor is referred to as *RV* (*for respiratory volume*). A time series representing the heart rate was calculated at each time point using the inverse of the average beat-to-beat interval over a 6 second sliding window. This was then convolved with the impulse response function for signal fluctuations related to heart rate (i.e. the ‘cardiac response function’) proposed in Chang et al.(2009) and sampled to the selected reference slice. The resulting regressor is referred to as *HR* (*for heart rate*).

Finally, the motion parameters estimated in the realignment step in SPM8 were used to create an additional set of 6 regressors (3 for translation and 3 for rotation) to represent a linear model for the residual effects of head motion after re-alignment (Friston et al., 1996), which we refer to below as *MP* (*for motion parameters*).

In summary, for each subject, a set of 20 regressors (i.e. CRP, RV, HR and MP) was calculated as described above for each EPI run. Noise correction based on different combinations of these regressors was studied.

### Analysis of temporal SNR dependence on image SNR

As an initial step, two regressors were constructed to model low frequency temporal drifts, nominally attributed to hardware effects, by creating a linear and quadratic function of image number (referred to as *HW for hardware*). Each of the five time series acquired at different flip angles was corrected for the low frequency drifts alone and also in addition to the different physiological effects described by the models above. The correction was performed by fitting the regressors (after mean correction) to the realigned and distortion corrected data using a GLM and subtracting the fitted effect from the image intensity. The data were corrected using the following combinations of models: 1) HW, 2) HW & CRP, 3) HW & RV & HR, 4) HW & CRP & RV & HR, 5) HW & MP, and 6) HW & CRP & RV & HR & MP, i.e., all models. These model combinations were selected to investigate the most commonly used approaches to noise correction (i.e. correcting for cardiac and respiratory phases (Glover et al., 2000), lower frequency changes in respiration and heart rate (Birn et al., 2006, 2008; Chang et al., 2009; Shmueli et al., 2007) and residual effects of motion and the combination of these effects with and without correcting for residual effects of head motion (Friston et al., 1996)).

The tSNR was defined as the mean of a voxel time series divided by its standard deviation. The image SNR was defined as the mean of the voxel time series divided by a measure of thermal noise from the images acquired with no RF excitation (see Appendix A). This method was shown to accurately reflect the true image SNR (SNR_0_) for multi-channel receiver coils with no noise covariance between receivers (Constantinides et al., 1997). Since we cannot guarantee the absence of noise covariance in our multi-channel receiver coil, we define SNR′_0_ as the measured image SNR and a scale factor κ such that SNR′_0_ = κ SNR_0_. The scale factor κ therefore accounts for noise covariance and relates true image SNR_0_ to the SNR′_0_ measure proposed by Constantinides et al.(1997). Finally, the mean tSNR and mean SNR′_0_ were calculated for voxels within the VC and LGN ROIs and averaged over subjects for the time series after correction for the different physiological effects. Note that no smoothing was applied to the images for consistency with other studies of tSNR (e.g. Kruger and Glover, 2001; Triantafyllou et al., 2005). An extension of the SNR model proposed in Kruger and Glover(2001) (see Appendix A and Hutton et al., submitted for publication for details) was fitted to the resulting mean tSNR as a function of mean SNR′_0_ using a multidimensional unconstrained nonlinear minimization (Nelder–Mead) method in Matlab. This resulted in an estimate of the model parameters 1/λ and κ for the data after being corrected using the different noise models (1 to 6). The different values of the parameter 1/λ can be considered to give an indication of the point at which the tSNR is degraded by further signal-dependent fluctuations (i.e. those not explained by the corresponding noise correction model). These values were also compared with those in the literature reported for data acquired at 3 T and 7 T and with different voxel sizes (Kruger and Glover, 2001; Triantafyllou et al., 2005).

Subject specific maps of the percent improvement in tSNR for models 2 to 6 compared with model 1 were calculated for the data acquired at the maximum flip angle (i.e. % ((tSNR_n_ / tSNR_1_) − 1) for n = model number = 2 to 6). Subject specific tSNR maps, percent improvement in tSNR maps and SNR′_0_ maps (for the maximum flip angle) were transformed into MNI space using the spatial normalization parameters calculated previously and averaged across subjects. These summary maps were used to demonstrate the spatial distribution of the impact of the different correction methods and the corresponding image SNR values.

### Performance of physiological noise correction for different spatial resolutions

The relative importance of the different physiological noise models was assessed by fitting GLMs comprising the different sets of physiological regressors to the realigned distortion corrected fMRI voxel time series acquired with the three different sets of acquisition parameters. Note that no smoothing was applied for this analysis. We fitted the six models investigated above (i.e. 1) HW, 2) HW & CRP, 3) HW & RV & HR, 4) HW & CRP & RV & HR, 5) HW & MP, 6) HW & CRP & RV & HR & MP) to each of the time series. To assess in more detail the importance of different noise components we also fitted six additional models for cardiac and respiratory phase components separately (i.e. 7) HW & CP and 8) HW & RP), respiratory volume and heart rate separately (i.e. 9) HW & RV and 10) HW & HR as well as 11) HW & CRP & MP and 12) HW & RV & HR & MP). All 12 GLMs additionally comprised regressors to model variance associated with the visual stimulus. The adjusted coefficient of determination (R^2^_adj_) was calculated at each voxel to compare the proportion of time series variance accounted for by each of the models while adjusting for the different numbers of regressors in each of the models. R^2^_adj_ is defined as R^2^_adj_ = 1 − (*SS*_*err*_ / *SS*_*tot*_) (*df*_*tot*_ / *df*_*err*_), where *SS*_*err*_ = (standard deviation of the residual errors)^2^, *SS*_*tot*_ = (standard deviation of time series)^2^, *df*_*tot*_ = number of degrees of freedom in the data − 1 and *df*_*err*_ = *df*_*tot*_ *−* 1 — number of degrees of freedom in each model. The resulting R^2^_adj_ values were averaged over voxels within the VC ROI and then over subjects.

To investigate the impact of the different physiological noise models on tSNR as a function of voxel volume, maps of tSNR were calculated for each time series acquired with the different parameter sets and after correction with models 1 to 12 as described above*.* The mean tSNR was calculated for voxels within the VC ROI and averaged over subjects.

### Analysis of fMRI study

The fMRI time series acquired for each subject were minimally spatially smoothed using an isotropic Gaussian kernel with FWHM = 2 mm for Acq1, 3 mm for Acq2 and 4 mm for Acq3. Six different GLMs were constructed comprising regressors to model variance associated with: 1) the visual stimulus alone or together with the physiological noise models, 2) CRP, 3) RV & HR, 4) CRP & RV & HR, 5) MP, and 6) CRP & RV & HR & MP. In each case, the GLMs were fitted to the data after high-pass filtering the time series using a cut-off period of 128 s. This step replaced the linear and quadratic detrending of the time series used for the analysis of temporal SNR dependence on image SNR (i.e. the model referred to as HW) and was performed to be in accordance with standard fMRI analysis procedures.

Voxel-wise t-tests were performed to detect BOLD effects that were greater for presentation of flickering left wedges compared to the blank screen and for right wedges compared to the blank screen. The number of significantly activated voxels (i.e. with p-value < 0.05 corrected for family-wise errors over the brain using random field theory, Worsley et al., 1996) was counted within the VC ROI (for all acquisitions) and LGN ROI (for Acq3 only) for models 1 to 6. The percentage difference in numbers of significantly activated voxels was calculated for models 2 to 6 compared with model 1 (i.e. between models using different physiological noise models compared to none) then averaged over subjects to provide an aggregate measure reflecting the change in BOLD sensitivity as a result of the noise correction model.

## Results

### Temporal SNR dependence on image SNR

**Fig. 1:**
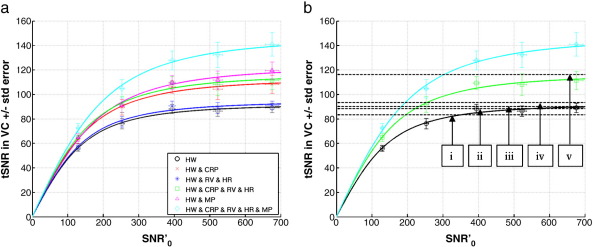
Temporal (tSNR) versus image SNR (SNR′_0_) in the visual cortex (VC). (a) Each data point represents the mean tSNR versus the mean SNR′_0_ in the VC ROI for flip angle = 8°, 16°, 26°, 38° and 70° ± standard error over 5 subjects. The different lines correspond to the fit of the extended SNR model (Hutton et al., submitted for publication; Kruger and Glover, 2001 and see Appendix A) to the tSNR and SNR′_0_ of the EPI time series after different noise correction models; 1) black circles — HW, 2) red crosses — HW & CRP, 3) dark blue stars — HW & RV & HR, 4) green squares — HW & CRP & RV & HR, 5) pink triangles — HW & MP, 6) light blue diamonds — HW & CRP & RV & HR & MP. Note that SNR′_0_ = κ SNR_0_ and κ ≈ 1.7. (b) Black horizontal dashed lines represent the 1/λ values from the literature; (i) 3 T data with voxel size ~ 41.36 mm^3^, 1/λ = 83.3 (Kruger and Glover, 2001), (ii) 7 T data with voxel sizes = 1 mm^3^ up to 75 mm^3^, 1/λ = 90.1, (iii) 3 T data with voxel sizes = 1 mm^3^ up to 75 mm^3^, 1/λ = 88.5, (iv) 3 T data with voxel size = 14.44 mm^3^, 1/λ = 93.5, (v) 7 T data, voxel size = 14.44 mm^3^, 1/λ = 116.3, (ii to v from Triantafyllou et al., 2005). The green line with squares, the light blue line with diamonds and the black solid line with circles are the same as those shown in (a).

Fig. 1a shows tSNR as a function of SNR′_0_ in the VC ROI when the image SNR was modulated by varying the flip angle. Each data point shows the mean and standard error over 5 subjects of the mean tSNR in the visual cortex (VC ROI). The different lines correspond to the fit of the extended SNR model (Hutton et al., submitted for publication; Kruger and Glover, 2001 and see Appendix A) to the tSNR and SNR′_0_ values averaged over the subjects after correction with different noise correction models; 1) black circles — HW, 2) red crosses — HW & CRP, 3) dark blue stars — HW & RV & HR, 4) green squares — HW & CRP & RV & HR, 5) pink triangles — HW & MP, and 6) light blue diamonds — HW & CRP & RV & HR & MP. The straight black dotted line shows tSNR = SNR′_0_ (i.e. in the absence of any additional temporal instability).

The mean and standard error of the SNR′_0_ and tSNR for the maximum excitation flip angle (70°) in the VC ROI averaged over the subjects for different noise correction models are shown in Table 2 as well as the mean and standard error of the model fit parameter 1/λ estimated from the model fits to the data from each subject corrected with different noise correction models. Note that these values are similar to the tSNR values estimated at the maximum image SNR values measured in this study. The maximum tSNR values for models (2) to (6) were compared to that for model (1) to calculate a percent improvement in tSNR that could be attributed to using the corresponding noise model (shown in Table 2). The values estimated for the model fit parameter κ were 1.7, 1.6 and 1.8 (mean, minimum and maximum respectively) over all subjects and all physiological noise models (also shown in Table 2).

Not surprisingly the largest improvements were observed when the noise correction model included all cardio-respiratory components and motion parameters (model (6)). The model including motion parameters alone (model (5)) showed a larger improvement than the model for all cardio-respiratory effects but no motion parameters (i.e. model (4)). The improvements due to the model including cardio-respiratory effects could be mainly attributed to the cardiac and respiratory phase components (i.e. compare models (2) and (3)). At lower SNR′_0_ values, e.g. below 200, the tSNR improvement was ~ 43% for model (6) and ~ 22% for model (4) (see Fig. 1a).

Fig. 1b shows 1/λ values reported in the literature (see figure legend for references) corresponding to the maximum achievable tSNR values for these studies. The solid black line with circles, the green line with squares and the light blue line with diamonds are the same as those in Fig. 1a and are shown for comparison. There was an overall consistency between the literature values estimated for both 3 T and 7 T data (dashed lines labeled i to iv) and the value estimated in this study for model (1) (i.e. no correction for physiological artifacts). The maximal tSNR value shown by the dashed line labeled (v) was of the same order as that estimated by models (2), (4) and (5) in this study (only (4) is shown), but still lower than that estimated for model (6). It is important to note that the model fit parameters represented by the dashed lines i, iv and v were predicted using data acquired at lower image SNR values below 200 (e.g. see Fig. 1 in Triantafyllou et al., 2005) so that the resulting 1/λ values which are greater than 100 were predicted from the model rather than measured for higher image SNR values.

**Fig. 2:**
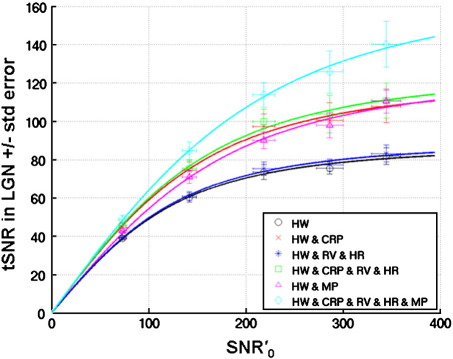
Temporal (tSNR) versus image SNR (SNR′_0_) in the lateral geniculate nucleus (LGN). Each data point represents the mean tSNR versus the mean SNR′_0_ in the LGN ROI for flip angle = 8°, 16°, 26°, 38° and 70° ± standard error over 5 subjects. The different lines correspond to the fit of the extended SNR model (Hutton et al., submitted for publication; Kruger and Glover, 2001 and see Appendix A) to the tSNR and SNR′_0_ of the EPI time series after different noise correction models; 1) black circles — HW, 2) red crosses — HW & CRP, 3) dark blue stars — HW & RV & HR, 4) green squares — HW & CRP & RV & HR, 5) pink triangles — HW & MP, 6) light blue diamonds — HW & CRP & RV & HR & MP. Note that SNR′_0_ = κ SNR_0_ and κ ≈ 1.6.

Similar to Fig. 1a, Fig. 2 shows tSNR as a function of SNR′_0_ in the LGN. Table 2 shows the mean and standard error of the SNR′_0_ and tSNR for the maximum excitation flip angle (70°), the mean and standard error of the model fit parameter 1/λ, the mean, minimum and maximum over models of the model fit parameter κ and the percent improvement in tSNR in the LGN ROI averaged over subjects for the different noise correction models. Notably, in the LGN ROI, the improvement using correction model (6) could be equally attributed to modeling cardio-respiratory effects and motion effects, whereas for the VC ROI more of the improvement using model (6) could be attributed to modeling the motion effects. This could be explained by sub-cortical regions being less sensitive to the effects of head motion than more superficially located brain regions such as the visual cortex, which may be more affected by the inhomogeneous coil sensitivity profile and contrast edges.

**Fig. 3:**
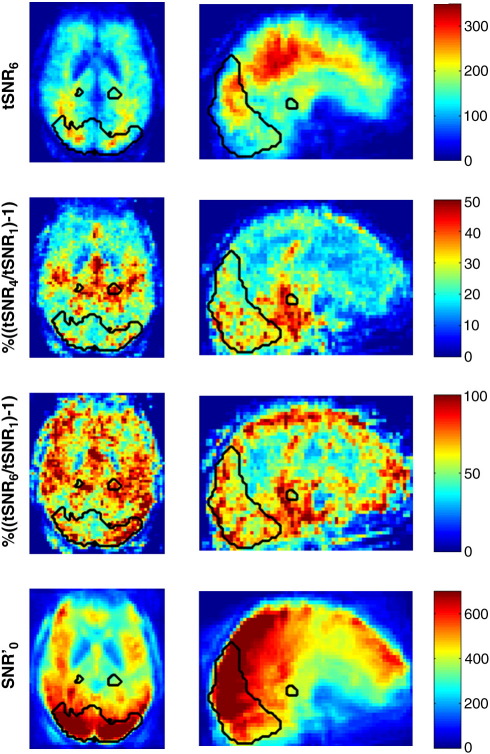
Maps of tSNR, percent improvement in tSNR and SNR′_0_ spatially normalized into MNI space and averaged over 5 subjects. Results are shown for data acquired with flip angle = 70°. Top row: tSNR map after physiological noise correction model (6) (HW & CRP & RV & HR & MP). Note the higher tSNR in the white matter. Second row: percent improvement in tSNR calculated after physiological noise correction model (4) compared to model (1): % ((tSNR_4_ / tSNR_1_) − 1). Third row: percent improvement in tSNR calculated after physiological noise correction models (6) compared to model (1): % ((tSNR_6_ / tSNR_1_) − 1). Bottom row: SNR′_0_ map where SNR′_0_ = κ SNR_0_. Note that this is the same for all correction models. The black contours illustrate the boundaries of the VC and LGN ROIs in MNI space rather than the actual subject specific gray matter ROIs used to analyze the tSNR and SNR′_0_ values. Note the large improvement in tSNR for % ((tSNR_6_ / tSNR_1_) − 1) especially in the VC ROI and around the edges of the brain whereas for % ((tSNR_4_ / tSNR_1_) − 1) the improvements are more specifically located in regions typically associated with physiological effects such as the regions of high vasculature.

Furthermore, in contrast to the VC ROI results, the model fit parameters for models (4) and (6) predict maximal values for tSNR that are greater than those actually measured (i.e. for model (4), 1/λ = 127.8 whereas the maximum measured tSNR is 110.8 and for model (6), 1/λ = 174.3 compared with the measured value of 140.2). This could be attributed to the use of the 24-channel receive head coil which has a high sensitivity and offers high image SNR at the surface of the brain compared with sub-cortical regions (compare the SNR′_0_ in the LGN with that in the VC ROI, as can be seen in the bottom row of Fig. 3). Thus the limited image SNR in the LGN may have prevented the predicted maximal tSNR from being obtained. The values estimated for the model fit parameter κ were 1.6, 1.4 and 1.7 (mean, minimum and maximum respectively) over all subjects and all physiological noise models (also shown in Table 2).

Fig. 3 shows maps of percent improvement in tSNR averaged over subjects. In the top row, the spatially normalized averaged map of tSNR for model 6 shows the pattern commonly reported in the literature for higher tSNR values in white matter compared to gray matter. The second and third rows show the percent improvement in tSNR for model (4) and model (6) respectively compared with model (1) (note the different color scales). For both maps, larger improvements in tSNR can be observed in regions typically associated with physiological effects such as highly vascularized regions. Additionally, for model (6) large improvements in tSNR can be observed around the edges of the brain and the VC ROI reflecting the impact of including motion parameters for correcting motion artifacts in these regions. The bottom row of Fig. 3 shows the spatially normalized averaged map of SNR′_0_ for comparison with the tSNR and improvement in tSNR maps.

### Performance of physiological noise correction for different spatial resolutions

**Fig. 4:**
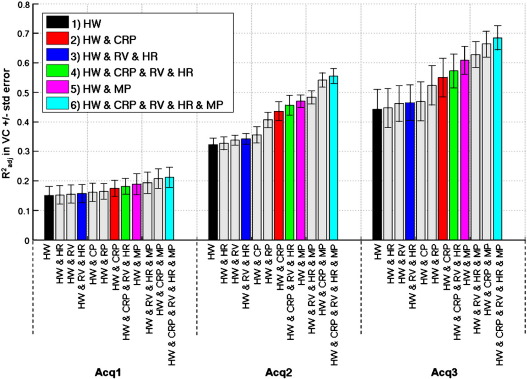
Adjusted coefficient of determination (R^2^_adj_) averaged over VC ROI ± standard error over 5 subjects for three different sets of acquisition parameters, Acq1, Acq2 and Acq3 (see Table 1 for acquisition parameters). The colored bars correspond to the model combinations 1 to 6 (see Fig. 1a) and the gray bars correspond to the additional models studied for completeness (as listed in horizontal axes).

Fig. 4 shows the R^2^_adj_ values in ascending order for 12 different physiological noise models for each of the three different sets of fMRI acquisition parameters. Each bar shows the mean and standard error over 5 subjects in the VC ROI. The colored bars correspond to the six model combinations investigated for all studies and the gray bars correspond to the additional six models studied for completeness. Using the R^2^_adj_ value means that the proportion of variance explained by each model can be compared even though the numbers of regressors are different between the models. For all acquisitions R^2^_adj_ was greatest for model 6 even though it comprised the most regressors. The order of R^2^_adj_ values for the more common combinations of models corresponded to the order of tSNR improvement in Fig. 1a and for all models the order was consistent for all acquisitions. Investigating physiological components separately showed that changes in respiration (RV) explained slightly more variance than changes in heart rate (HR), respiratory phase (RP) explained slightly more variance than the cardiac phase (CP) and cardiac and respiratory phase components together explained more variance than changes in heart rate and respiration. The most variance was explained by all the models containing the motion parameters with the motion parameters explaining the largest portion of the variance. For Acq1 and Acq2 the models for cardiac and respiratory phases and also for motion parameters each explained more than an additional 10% of variance. Furthermore the model comprising all effects explained more than an additional 20% of the variance even though this model included all 20 regressors. The overall reduction in R^2^_adj_ values with decrease in voxel size reflected the reduced contribution of physiological noise at lower image SNR values.

**Fig. 5:**
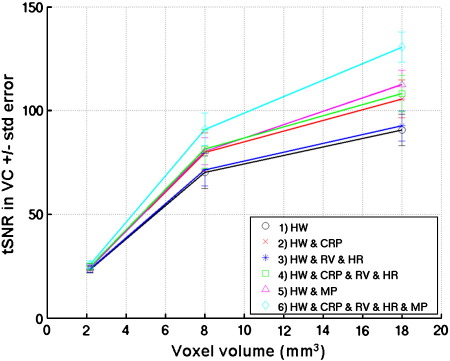
Temporal (tSNR) versus voxel size (in mm^3^) for three sets of acquisition parameters (Acq1, Acq2 and Acq3 respectively, see Table 1 for acquisition parameters). Each data point represents the mean tSNR in the VC ROI ± standard error over 5 subjects. The different lines correspond to the tSNR of the EPI time series after different noise correction models; 1) black circles — HW, 2) red crosses — HW & CRP, 3) dark blue stars — HW & RV & HR, 4) green squares — HW & CRP & RV & HR, 5) pink triangles — HW & MP, 6) light blue diamonds — HW & CRP & RV & MP.

Fig. 5 shows the tSNR as a function of voxel size for different physiological noise models. Note that only models 1 to 6 are shown for clarity. Each data point shows the mean and standard error over 5 subjects of the mean tSNR in the visual cortex (VC ROI). Compared to model (1) the maximum percent improvement in tSNR was around 10% for Acq1, 30% for Acq2 and 40% for Acq3. There was a reduction in tSNR improvement as the voxel size was reduced which again reflected the reduced contribution of physiological noise as a result of the lower image SNR for the increased spatial resolution. Note that the results in Figs. 4 and 5 were generated from EPI time series during which visual activation studies were performed.

### fMRI study

**Fig. 6:**
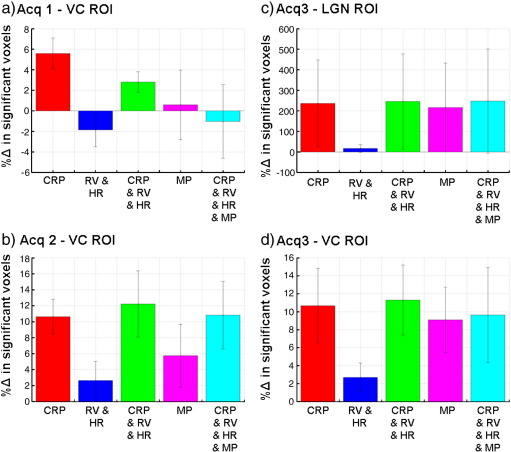
Percent difference in numbers of significantly activated voxels (p < 0.05 corrected for multiple comparisons) for physiological noise correction models (2) to (4) compared with model (1): 2) red — CRP, 3) dark blue — RV & HR, 4) green — CRP & RV & HR, 5) pink — MP and 6) light blue — CRP & RV & HR & MP. Each bar represents the results averaged over subjects ± standard error. The different panels show results for the different sets of acquisition parameters and ROIs — a) Acq1, VC ROI, b) Acq2, VC ROI, c) Acq3, LGN ROI and d) Acq4, VC ROI (see Table 1 for acquisition parameters).

Fig. 6 shows the percent difference in numbers of significantly activated voxels (p < 0.05 corrected for multiple comparisons) for the fMRI analysis calculated for physiological noise correction models (2) to (4) compared with model (1) averaged over subjects. For Acq2 and Acq3 all noise models lead to an increase in the number of significantly activated voxels when averaged over subjects. For all acquisitions the cardiac and respiratory phase models (2) and (4) always lead to improvements (> 10% for Acq2 and Acq3 in the VC ROI). Models including motion parameters lead to improvements for Acq2 and Acq3 but to a lesser degree. The model for lower frequency cardio-respiratory effects alone (model (3)) resulted in the smallest change in numbers of significantly activated voxels and even lead to a small reduction in the VC ROI for Acq1. In the LGN ROI for Acq3, average increases of more than 200% were observed for all models except model 3, which reflects the small number of activated voxels in this region. The standard errors indicated that the range of improvements was highly variable across subjects. This was also apparent when examining the activation maps from individual subjects. For example in one subject, LGN activation was only present in one hemisphere when no physiological noise modeling was included in the GLM (i.e. model (1)), but became significantly activated after noise correction was performed.

## Discussion

The goals of this study were to characterize the effect of physiological noise correction on temporal SNR (tSNR) as a function of image SNR and to investigate the impact of these corrections on BOLD sensitivity. We placed emphasis on fast whole brain image acquisition parameters that are typically used for cognitive neuroimaging studies. We acquired task-free EPI time series at different radio-frequency (RF) excitation flip angles, applied physiological noise correction methods and fitted a modified version of the SNR model proposed by Kruger and Glover(2001) to the tSNR and SNR values resulting from each time series. We also performed a visual activation fMRI study at different spatial resolutions and assessed changes in the numbers of significantly activated voxels associated with brain activity after the different correction methods. Using state of the art models for the physiological noise correction, we showed increases in tSNR per unit time beyond previously published values and related improvements in BOLD sensitivity.

### Performance of different physiological noise models

By removing the variance in the EPI time series associated with the different physiological noise components it was possible to improve the tSNR by approximately 25% in the visual cortex and 35% in the LGN when including regressors for cardio-respiratory effects. When additionally including the motion parameters the tSNR was improved by approximately 58% in the visual cortex and 71% in the LGN. Overall, the largest portion of variance was explained by the motion parameters. This would suggest that a large amount of the variance was due to subject motion. On inspection, the estimated motion parameters indicated that the subjects did not move excessively (less than 1 mm translation and 1° rotation), but it was apparent in the physiological noise model that the estimated motion parameters were partially correlated with subject breathing. Respiration may lead to small true movements of the head, as well as a modulation of the B_0_ magnetic field which has been shown to cause significant fluctuations at 7 T (Van De Moortele et al., 2002). As the magnetic field changes, geometric distortions leading to voxel shifts along the phase-encode direction also fluctuate. This can cause the retrospective image realignment algorithm to make an incorrect estimate of true head motion along the phase-encode direction. This effect was apparent in the estimated motion parameters for the data acquired in this study. One approach to mitigate this effect is the real-time adjustment of B_0_ shims (van Gelderen et al., 2007) which uses information about the spatial distribution of the magnetic field changes associated with breathing to apply compensating B_0_ shims in real time. Such methods require special hardware and are not readily available. Image-based methods to retrospectively correct for fluctuating geometric distortion effects have been proposed (Andersson et al., 2001; Hutton et al., 2005) but currently use models based on true head motion which would need to be modified to include information about the subject's respiration.

Corrections based on the model for cardiac and respiratory phase changes had a much greater impact on the tSNR than the model for lower frequency cardio-respiratory effects (e.g. at the maximum image SNR, tSNR was improved by ~ 22% compared with ~ 2% in the VC ROI and ~ 32% compared with ~ 2% in the LGN). One explanation is that changes in the breathing and heart rates of the subjects studied here were minimal. In contrast, the models for cardiac and respiratory phases account for much of the variance attributed to the sampling of the cardiac and respiratory phases at different time points.

The performance of the corrections was most dramatic for the data acquired at the lowest spatial resolution but still had a significant effect for the medium spatial resolution. The order of performance of the different models was comparable at different resolutions, in particular, the model including all regressors always performed best. At the smallest voxel size, improvements were minimal, reflecting the reduced contribution of physiological noise as a result of lower image SNR.

### Comparison with other studies on physiological noise and temporal SNR

In recent work we have extended the model proposed in Kruger and Glover(2001) to account for noise covariance in data acquired using a multi-channel receiver coil (see Appendix A and Hutton et al., submitted for publication). By fitting this extended model to tSNR values calculated for different SNR′_0_ values, it was possible to estimate the parameters 1/λ and κ for the data after different physiological noise correction models were applied. The parameter κ is a scale factor which represents the deviation of the SNR′_0_ measured using the method introduced by Constantinides et al.(1997) from the true SNR_0_ and reflects the impact of noise covariance on the data as a result of the multi-channel receiver coil (i.e. κ = SNR′_0_ / SNR_0_). The parameter 1/λ can be considered as a physical measure of tSNR degradation by signal dependent fluctuations. In other words it provides an indication of the tSNR at which further increases in image SNR do not lead to further increases in tSNR. In the literature, this parameter has been estimated for data acquired at different field strengths and different voxel sizes (Kruger and Glover, 2001; Triantafyllou et al., 2005). In order to fit the model, image SNR was modulated by varying the flip angle (as was done in this study) or by varying the voxel size. The published 1/λ values (i to iv in Fig. 1b, from Kruger and Glover, 2001 and Triantafyllou et al., 2005) are quite consistent with the data acquired in this study after applying the correction using the less effective models ((1) and (3)). The exception is the case of the 7 T data acquired with a smaller voxel size of 14.44 mm^3^, for which 1/λ = 116.3 (dashed line v in Fig. 1b). In this case it was shown that the reduction in image SNR resulting from the increased resolution beneficially leads to a reduction in the relative contribution of physiological noise to tSNR (Triantafyllou et al., 2005). It should also be noted that the maximum tSNR measured for this data was below 100, so as pointed out by the authors, obtaining data with a tSNR equal to 1/λ would require the image SNR to be increased using improved RF coil design or even higher field strengths. Furthermore, at higher field strengths it is also important to account for large B1 inhomogeneities (Lutti et al., 2010) when setting the RF excitation flip angle in order to maximize the image SNR in a particular region of interest. In this experiment the scanner adjustment procedures resulted in an actual RF excitation flip angle which was reasonably close (within ~ 10%) of the nominal flip angle in the visual cortex.

The higher SNR available at 7 T can be exploited to acquire high resolution data (Speck et al., 2008) and the corresponding reduction in image SNR has been shown to reduce the relative contribution of physiological noise (Triantafyllou et al., 2005). Furthermore, by acquiring high resolution data and then spatially smoothing it down to a lower resolution, the tSNR can be increased relative to that achieved by direct acquisition of the same spatial resolution (Triantafyllou et al., 2006). For example, for data acquired with an in-plane resolution of 1.5 mm × 1.5 mm, then smoothed down to a resolution of 3 mm × 3 mm, the ratio of tSNR of the smoothed data to that of the data directly acquired at the same lower spatial resolution was 1.61 (Triantafyllou et al., 2006). This tSNR improvement is of the same order as the maximum improvement achieved in this study for data acquired with an in-plane resolution of 3 mm × 3 mm (i.e. a 58% to 71% improvement in tSNR).

Although there are benefits in high resolution acquisition, there are also limitations. In particular, temporal resolution is reduced and susceptibility related geometric distortions are exacerbated due to longer readout times. Furthermore, increased spatial resolution can lead to reduced brain coverage for the same TR (e.g. the LGN was not imaged in all subjects for the higher resolution acquisitions presented in this study). The longer acquisition time will also reduce the number of observations in an fMRI experiment and hence the overall sensitivity of the experiment (Murphy et al., 2007). To take this into account, the comparison of tSNR achieved using different protocols should include the effective degrees of freedom or volume acquisition time, for example using the measure for ‘sensitivity’ proposed in Poser et al.(2010), tSNR/√volume TR. It should be noted that the temporal autocorrelations in fMRI time series occur at a time scale of a few seconds so that a shorter TR may lead to a slightly higher autocorrelation affecting the sensitivity measure. The ratio between the minimum √volume TR proposed in Triantafyllou et al.(2006) for high resolution acquisition at 7 T and the lowest resolution used in the current study for the same brain coverage suggests an improvement in sensitivity for the current study by a factor of √3/2.25 assuming that the temporal autocorrelations are equivalent in the two acquisitions. In other words, the high resolution experiment would need to be 33% longer to achieve the same statistical power as the experiment using a standard resolution with physiological noise correction. In a conservative estimate where the autocorrelation is 0.1 for the high resolution acquisition and 0.2 (Penny et al., 2003) for the low resolution scan, the experiment would still need to be 10% longer for the high resolution acquisition. It should be noted that comparisons between scanners and protocols may also be affected and possibly limited by differences in the RF coils, k-space acquisition schemes, image reconstruction methods and timing parameters such as TE.

The results presented here show that it is possible to increase the tSNR for EPI acquired at a lower resolution using physiological noise correction methods and without sacrificing imaging speed, coverage or image SNR. However, it is also clear that the increase in tSNR relative to the maximum available SNR is still limited (i.e. 140 to 400). Possible explanations for at least some of this residual variance are spontaneous neuronal fluctuations (Bianciardi et al., 2009) or signal fluctuations generated as a result of the image reconstruction. Nevertheless, the tSNR achieved with the full noise correction model (6) exceeds values for tSNR per unit time reported in the literature so far (Bodurka et al., 2007; Gonzalez-Castillo et al., 2011; Hu and Glover, 2006; Kruger and Glover, 2001; Murphy et al., 2007; Triantafyllou et al., 2005, 2006, 2011) as determined by a systematic literature search (search of the PubMed database for titles and abstracts containing the terms “physiological noise AND SNR AND (MRI OR fMRI OR 7 T)” followed by manual selection of relevant articles (8 out of 10)).

### Performance of physiological noise correction in fMRI

The results of the visual fMRI study showed that for all acquisitions physiological noise correction using the cardiac and respiratory phase models leads to an increase in the number of significantly activated voxels of more than 10% in the visual cortex and more than 200% in the LGN. In agreement with the tSNR analyses, the model for lower frequency cardio-respiratory effects alone resulted in the smallest change in numbers of significantly activated voxels and overall the largest improvements were seen for data acquired using Acq3 and in the LGN ROI.

The relative improvements observed in the analysis of tSNR did not completely transfer to improvements in BOLD sensitivity as assessed by an increase in significantly activated voxels. In fact, in some subjects the corrections which included the motion parameters even had a detrimental impact BOLD sensitivity. This effect suggests that including the motion parameters to model residual errors may have reduced the size of the modeled visual response which can happen if components of the noise models were correlated with the activation task. On closer inspection of the GLM, correlations between the head motion parameters and task were higher for subjects where this effect was apparent. This may have been caused by actual task-related head motion leading to false positives which are removed by the correction. It is also possible that large signal changes from the visual activity introduced a bias into the retrospective image realignment algorithm hence resulting in artifactual task-correlated motion estimates as described by Freire and Mangin(2001). This issue could be resolved by estimating the motion from independent measurements such as optical tracking (Zaitsev et al., 2006) or by developing fully quantitative artifact models that do not rely on a correlational approach.

Discrepancies between the tSNR and fMRI results may have been caused by the difference in the standard methods used for each analysis to perform physiological noise correction. The analyses were performed in this way so that improvements in tSNR calculated using a standard method (Friedman and Glover, 2006) could be compared with increases in BOLD sensitivity measured using standard fMRI analysis methods (Friston, 2007). For the tSNR study, no smoothing was applied to the data before fitting the GLM and linear and quadratic functions of image number were used to model low frequency drifts. For the fMRI study, data were smoothed with an isotropic Gaussian kernel with FWHM = 2 mm, 3 mm and 4 mm for Acq1, Acq2 and Acq3 respectively before fitting the GLM and a high-pass filter was used to remove low frequency drifts. Furthermore, for the statistical analysis of fMRI data, the structured non-white noise or temporal autocorrelations resulting from physiological effects can lead to invalid statistical inferences. Most fMRI analysis software packages try to compensate for this using for example an autoregressive (AR) model (Friston et al., 2002). However it has been shown that a first order AR model is inadequate in the presence of strongly correlated oscillatory noise and that specific modeling of physiological effects was a superior approach for improving the validity of statistical inferences (Lund et al., 2006). Thus, in general, the direct comparability of different statistical models may be limited, since the analyses take oscillatory noise components into account to different degrees. Finally, summarizing changes in BOLD sensitivity over a region of interest becomes complicated when one considers that the differential effects of the noise correction methods as described above also vary from voxel to voxel. As a result the aggregate measure used to represent the within subject changes in BOLD sensitivity may contribute to the between subject variability.

### Methodological considerations

The methods used in this study can be implemented easily for routine fMRI scanning and data analysis. Peripheral measurements of subject pulse and breathing are straight forward, even in the MRI environment, and should not interfere with experimental design. Although a time lag may exist between the pulse measured at the finger tip and that measured in the head, the oscillatory nature of the correction means that the lag will result in a phase shift only. The cardiac modeling in the cardiac respiratory phase (CRP) model (Glover et al., 2000) is by design insensitive to phase shifts. However, the heart rate (HR) model may in principle be affected by such a lag, which may explain to some degree why this model is less effective in reducing physiological noise in the data. Peripheral physiological monitoring may also be further simplified by capturing multiple sources of physiological noise using pulse oximetry alone (Verstynen and Deshpande, 2011).

The physiological noise correction methods described here will reduce the degrees of freedom in the data. For fMRI studies, the number of degrees of freedom is usually quite large (i.e. often comprising more than 100–200 images). Therefore modeling residual variance with up to 20 regressors (i.e. up to 10% reduction in degrees of freedom) will only minimally affect the statistical analysis. Furthermore, when the physiological regressors are included in the fMRI statistical model, the established statistical analysis techniques (e.g. as implemented in SPM8) will account for changes in the spatio-temporal variance structure and effective degrees of freedom (Friston, 2007).

In this study the methods used to extract the ROIs were carefully designed so that anatomical regions defined in MNI space were non-linearly transformed into subject-specific space and masked by gray matter voxels identified using segmentation. This ensured that comparable regions were automatically defined in all subjects and that the regions contained gray matter only to avoid tSNR values being contaminated by those from white matter voxels. Note that in this study we did not specifically investigate improvements in tSNR in brain regions suffering from susceptibility related distortion and signal loss. Although well-established methods exist to address these issues e.g. (Deichmann et al., 2002, 2003; Weiskopf et al., 2006, 2007), they need to be validated at 7 T.

## Conclusion

This study has demonstrated the impact of physiological noise correction on EPI time series acquired at 7 T. Modeling cardiac and respiratory effects in fMRI analyses is strongly recommended to improve BOLD sensitivity. To improve the tSNR, the largest proportion of residual variance was explained by including motion parameters, but for fMRI analyses care must be taken that the estimated head motion is not correlated with the fMRI task. Using the full noise models the tSNR was increased by more than 50–70% and in fMRI experiments the number of significantly activated voxels was increased by more than 10% in the visual cortex or much more sub-cortically. In summary, fMRI at 7 T in combination with optimized physiological noise modeling promises a very high BOLD sensitivity and temporal resolution for cognitive neuroimaging.

## Figures and Tables

**Table 1:** Acquisition parameters used for EPI data collection. For all acquisitions, TE = 25 ms and volume TR = 2.

	In-plane resolution (mm^2^)	Matrix	Slice thickness (mm)/gap	Number of slices	Readout BW (Hz/px)	Echo spacing (ms)	GRAPPA acceleration factor	Partial Fourier
Acq1	1.1 × 1.1	192 × 192	1.8/0	31	1530	0.76	3	7/8
Acq2	2 × 2	106 × 106	2/0	31	2245	0.51	–	6/8
Acq3	3 × 3	64 × 64	2/1	40	2298	0.5	–	–

**Table 2:** Results from the temporal SNR and fMRI studies for 6 different physiological noise correction models averaged over two different regions of interest and 5 subjects. The values shown are the mean and standard error of the SNR′_0_ (SNR′_0_ (70°)) and tSNR (tSNR (70°)) for the maximum excitation flip angle (70°), the mean and standard error over subjects of the model fit parameter (1/λ), the mean, minimum and maximum over models of the model fit parameter κ, percent improvement in tSNR (ΔtSNR (70°)) and percent increase in significantly activated voxels (ΔBOLD) for each model compared with model 1. Note that the SNR′_0_ (70°) values are the same for each model since the model regressors are mean corrected and SNR′_0_ = κ SNR_0_.

Results for visual cortex (VC ROI)					
Models	SNR′_0_ (70°)	tSNR (70°)	1/λ	ΔtSNR (70°) (%)	ΔBOLD (%)
1) HW	675.8 ± 19.2	88.9 ± 3.8	92.5 ± 3.2	–	–
2) HW & CRP	108.2 ± 7.4	113.7 ± 7.1	21.3 ± 4.3	10.7 + 4.2
3) HW & RV & HR	91.0 ± 3.6	95.0 ± 3.2	2.4 ± 0.5	2.7 + 1.6
4) HW & CRP & RV & HR	111.2 ± 7.4	117.3 ± 7.3	24.8 ± 4.4	11.3 + 3.9
5) HW & MP	119.5 ± 7.2	123.6 ± 7.1	34.5 ± 6.8	9.1 + 3.6
6) HW & CRP & RV & HR & MP	140.9 ± 9.6	147.6 ± 10.0	58.5 ± 8.7	9.6 + 5.3
[κ = 1.7/1.6/1.8 (mean/minimum/maximum across models)]					

*Results for lateral geniculate nucleus (LGN ROI)*					
1) HW	344.4 ± 15.1	81.8 ± 4.3	88.2 ± 3.6	–	–
2) HW & CRP	107.8 ± 8.5	123.2 ± 10.8	31.3 ± 4.5	236.0 + 211.4
3) HW & RV & HR	83.3 ± 4.5	90.3 ± 3.6	1.7 ± 0.3	16.5 + 19.8
4) HW & CRP & RV & HR	110.8 ± 9.1	127.8 ± 11.8	34.8 ± 5.1	245.4 + 231.6
5) HW & MP	110.6 ± 6.3	126.6 ± 9.1	35.6 ± 5.2	215.9 + 216.4
6) HW & CRP & RV & HR & MP	140.2 ± 11.9	174.3 ± 19.0	70.9 ± 8.5	247.6 + 253.4
[κ = 1.6/1.4/1.7 (mean/minimum/maximum across models)]					

## Appendix A Extended physiological noise model accounting for noise covariance in multi-channel receiver coil data

Using the model proposed by Kruger and Glover(2001) to characterize the effect of physiological noise correction on temporal SNR (tSNR) as a function of image SNR (SNR_0_) requires that tSNR and SNR_0_ are estimated accurately. In particular, the values should be identical when device instability and/or physiological noise are negligible (Triantafyllou et al., 2011). The tSNR is typically defined as the mean intensity of a voxel time series divided by its standard deviation and can be easily measured on magnitude time-series data. Accurately estimating the exact image SNR_0_ in principle needs to take into account the entire image acquisition and reconstruction process (Kellman and McVeigh, 2005). This includes for example noise covariance in multi-channel receiver systems, effective receiver bandwidth and noise rectification in magnitude images. To our knowledge, this is not implemented as standard image reconstruction on clinical scanners. Calculating the SNR_0_ off-line requires large amounts of k-space raw data from the individual channels and noise calibration scans. Therefore in this study we took an alternative approach to estimating SNR_0_ from the scanner vendor's root sum-of-squares (rSoS) reconstructed images. We used the approach by Constantinides et al.(1997), which requires an additional noise reference scan with no RF excitation but does not require raw k-space data. This approach computes the noise standard deviation σ = root mean square value of all the voxel intensities in the central region of the noise reference images divided by twice the number of receiver channels. At each voxel we then defined a measure, SNR′_0_ = mean intensity of the voxel time series divided by σ which was shown to accurately reflect SNR_0_ for multi-channel receiver coils with no noise covariance between receivers (Constantinides et al., 1997). In the presence of noise covariance between receivers, SNR′_0_ deviates from SNR_0_ by a scaling factor which reflects the impact of noise covariance on the data. Therefore to account for this we extended the model proposed by Kruger and Glover(2001) to include an additional scale factor κ such that:

(A1) $$\kappa ={{\text{SNR}}^{\prime }}_{0}/{\text{SNR}}_{0}$$

Using Eq. (A1) to replace SNR_0_ by SNR′_0_ in the original model proposed by Kruger and Glover(2001) leads to the extended model (Hutton et al., submitted for publication):

(A2) $$\text{tSNR}={{\text{SNR}}^{\prime }}_{0}/{\left(\kappa^{2}+\lambda^{2}{{\text{SNR}}^{\prime }}_{0}{}^{2}\right)}^{1/2}$$

This model enables the effect of noise covariance (κ) and physiological noise (λ) to be estimated simultaneously from readily available rSoS reconstructed images. Furthermore, the scaling factor κ may also capture other correction factors that are independent of image SNR. The λ and κ values estimated from the SNR′_0_ and tSNR values measured at different flip angles were estimated using a multidimensional unconstrained nonlinear minimization (Nelder–Mead) method in Matlab. Full details of the extended model and its validation using simulations, phantom and human data are described in Hutton et al.(submitted for publication). For small values of SNR_0_ (e.g. < 35 for the case of 24 receiver channels as used in this study), an additional correction of SNR′_0_ and tSNR is necessary to account for the changes in noise statistics. Note that in the absence of physiological noise or other temporal instabilities, e.g. by acquiring data on a phantom, the relationship tSNR = SNR′_0_/κ holds true.
